# Validation of a Real Time PCR for Classical Swine Fever Diagnosis

**DOI:** 10.1155/2014/171235

**Published:** 2014-04-09

**Authors:** Natanael Lamas Dias, Antônio Augusto Fonseca Júnior, Anapolino Macedo Oliveira, Érica Bravo Sales, Bruna Rios Coelho Alves, Fernanda Alves Dorella, Marcelo Fernandes Camargos

**Affiliations:** ^1^Laboratório de Biologia Molecular e Laboratório de Diagnóstico de Doenás Virais, Laboratório Nacional Agropecuário-Minas Gerais (LANAGRO-MG), Ministério da Agricultura, Pecuária e Abastecimento, Avenida Rômulo Joviano s/n, Fazenda Modelo, 33600-000 Pedro Leopoldo, MG, Brazil; ^2^Embrapa Dairy Cattle, Rua Eugênio do Nascimento 610, 36038-330 Juiz de Fora, MG, Brazil

## Abstract

The viral disease classical swine fever (CSF), caused by a *Pestivirus*, is one of the major causes of economic losses for pig farming. The aim of this work was to validate a RT-qPCR using Taqman for detection of CSF in swine tissues. The parameters for the validation followed the specifications of the Manual of Diagnostic Tests and Vaccines for Terrestrial Animals of the World Organization for Animal Health (OIE) and the guide ABNT NBR ISO/IEC 17025:2005. The analysis of the 5′NTR region of CSF virus was performed in 145 samples from 29 infected pigs and in 240 samples from 80 pigs originated in the Brazilian CSF-free zone. The tissues tested were spleen, kidney, blood, tonsils, and lymph nodes. Sequencing of the positive samples for 5′NTR region was performed to evaluate the specificity of the RT-qPCR. Tests performed for the RT-qPCR validation demonstrated that the PCR assay was efficient in detecting RNA from CSF virus in all materials from different tissues of infected animals. Furthermore, RNA from CSF virus was not detected in samples of swine originated from the Brazilian CSF-free zone. Hence, it is concluded that RT-qPCR can be used as a complementary diagnostic for CSF.

## 1. Introduction

Classical swine fever is a highly contagious disease that affects domestic and wild swine and is caused by a virus from Flaviviridae family and* Pestivirus* genre [1, 2]. The virus infection occurs through the oronasal and the first replication occurs in the tonsils. Further, the virus reaches the bloodstream and affects tissues such as lymph nodes, spleen, kidneys, distal ileum, and brain [3–6]. The implications of CSF include high animal mortality, reduction in productivity, and trade restrictions applied to the producing country. Hence, CSF is considered a major cause of economic losses to pig industries worldwide, including Brazil, where pig farming and industry holds nearly 600,000 direct and indirect jobs and ranks as the fourth world swine meat exporter [7].

The trade of live pigs and their byproducts in national and international scope represents a risk to areas or country free of CSF. Laboratory diagnosis of CSF is essential once many symptoms are not exclusively associated with the disease and may vary depending on the virus strain, age, and health status of the animals. Such diagnosis must be fast and accurate, especially in countries that has eradication programs already established, since the fast diagnosis helps reducing the potential for transmission for uninfected herds and therefore prevents the spread of the disease.

The gold standard diagnosis of CSF performed by virus isolation can be problematic, particularly due to biosecurity risks involved with handling live infectious agents. In addition, serological assays performed to diagnose CSF may yield confounding results because antibodies for other diseases caused by Pestiviruses, such as bovine viral diarrhea (BVDV) and border disease, can cross-react with the CSF virus [8, 9]. In order to overcome those caveats, a molecular assay can be performed as a complimentary test to serology for the diagnosis of CSF. The* Pestivirus *genome has a highly conserved nontranslated region (NTR), which has been amplified by various studies that performed polymerase chain reaction from reverse transcription of viral RNA (RT-PCR) [10–12]. Therefore, amplification of that* Pestivirus* NTR by RT-PCR represents a valuable complimentary alternative for CSF diagnosis. Advantages of using this molecular technique include the possibility of working with deteriorated samples, which otherwise is not possible when performing virus isolation [13], and the reduced biosecurity risk, once manipulation of active virus in cell culture for isolation is not required. In addition, the risk for incidents of cross-reactivity with other Pestiviruses, which is common in serological tests, is minimal by using this RT-PCR.

Thus, this study aimed to validate a RT-qPCR detection of the region 5′NTR of CSF virus from Brazilian swine. The validation of this assay was performed according to the recommendations of the Brazilian Society for Technical Standards [14] and included the comparison of the results from a standardized method (previously validated through collaborative studies) with recognized reference methods (official or international standard) followed by proper documentation of the performance of the method through proficiency test.

## 2. Material Studied, Area Descriptions, Methods, and Techniques

### 2.1. RT-qPCR Validation

In order to perform the validation of RT-qPCR detection of the region 5′NTR of CSF virus, samples from CSF-infected pigs (positive control) and from pigs originated from a CSF-free zone (negative control) were used. The CSF-infected pig samples were kindly provided by the National Agricultural Laboratory of Pernambuco (LANAGRO/PE, Ministry of Agriculture, Livestock and Supply), a laboratory located outside the Brazilian CSF-free zone and that routinely inoculates swine for tests of CSF vaccine potency. Twenty-nine pigs were intramuscularly infected with the Brescia strain of CSF virus and euthanasied three days later. At necropsy, a total of 145 samples of tissues including spleen, kidney, blood, tonsils, and a pool of lymph nodes were obtained from the CSF-infected pigs. The CSF-free zone samples were in total 240, comprising blood, tonsils, and kidney, from 80 pigs reared on Brazilian CSF-free zone and originated from a slaughterhouse certified by the Federal Inspection Service (SIF) and located in Minas Gerais, Brazil.

All samples were inactivated using TRIzol (Life Technologies, USA) before being sent to analysis. RNA was extracted from 100 mg of samples following the TRIzol protocol (Life Technologies, USA) according to the manufacturer's instructions. The concentration of the extracted material was estimated by spectrophotometer (NanoVue, GE Healthcare, UK).

Samples were subjected to a RT-qPCR to the normalizing gene beta-actin, following the protocol described by Bielanski et al. [15] in order to verify the efficiency of RNA extraction and the presence of inhibitors.

NTR region was chosen as the target for RT-qPCR, according to Hoffmann et al. [12] and as described in the Manual of Diagnostic Tests and Vaccines for Terrestrial Animals of the World Organization for Animal Health [16] with modifications. The reaction was performed using the QuantiFast Probe RT-PCR kit (Qiagen, Germany) and specific primers and probe [12]. The primers used were CSF 100-F: 5′ATGCCCAYAGTA GGACTAGCA3′ and CSF 192-R: 5′CTACTGACGACTGTCCTGTAC3′, and the probe was CSF Taqman FAM/BHQ1: TGGCGA GCTCCCTGGGTGGTCTAAGT. The master mix was composed of 2.25 µL RNase free-water; 12.5 µL 2X QuantiFast Probe RT-PCR Master Mix; 2 µL of each primer (10 µmol/µL); 0.5 µL of probe (10 µmol/µL); 0.25 µL Quantifast RT Mix, 0.5 µL of 50X Rox; 2 µL of RNA. The thermal profile was as follows: 50°C for 30 min, 95°C for 15 min, 45 cycles of 95°C for 10 sec, and 57°C for 1 min (ON-FAM). All reactions were performed in ABI7500 thermocycler (Life Technologies, USA).

### 2.2. Specificity

To evaluate the specificity of the test, nine samples of different genotypes were analyzed. The strains were CSFV RNA Alfort187 strain (genotype 1.1, reference strain); Brescia strain (genotype 1.2, reference strain); CSF0653 strain (genotype 1.3, Honduras 1992); Paderborn strain (genotype 2.1, Germany 1997 strain); CSF0849 strain (genotype 2.1, South Africa 2005); Parma98 strain (genotype 2.2, reference strain); Spain 2001 strain (genotype 2.3); CSF1045 strain (genotype 2.3, Germany 2009); CSF0864 strain (genotype 2.3, Bulgary 2007). The samples were obtained from the reference laboratory for CSF diagnosis, Instituto Nacional de Investigación y Tecnologia Agrária y Alimentaria, located in Spain. RT-qPCR products were sequenced in an automated sequencer ABI Prism 3130 Genetic Analyzer (Applied Biosystems, Foster City, CA, USA) using Big Dye Terminator Kit from the same manufacturers. Specific primers for the sequencing are CSF 52F: CCCTGGGTGGTCTAAGTCCT and CSF 281R: CTCCATGTGCAATGTACARC. Sequences were edited in Bioedit program [17] and compared to those published in GenBank, using Nucleotide Blast [18].

Specificity was also evaluated after the sequencing of 12 clinical samples from an outbreak of CSF that occurred in 2009, Rio Grande do Norte, Brazil. The analytical specificity of the RT-qPCR was checked by the absence of amplification of RNA of the BVDV-I NADL cytopathic strain, the BVDV-I W2 noncytopathic strain, viral DNA from the Paracambi strain of the African swine fever virus (ASF), and the viral DNA from Shope strain of the pseudorabies virus. All these viruses were growth in MDBK and PK15 cell lines tested for contamination as described by Pinheiro de Oliveira et al. [19].

### 2.3. Limit of Detection (LD)

Determination of the LD (analytical sensitivity) was performed using a blood sample collected from a CSFV infected swine. This sample had 10^5.8^ TCID_50_/mL titer, and it was serially diluted until 10^−6^. Each dilution was tested in triplicate and the concentration was estimated by spectrophotometer UV light (NanoVue, GE Healthcare, US). The assay was repeated in three different days. Standard curves were made for the determination of the optimal concentration of primers, defining 0.99 as minimum *R* ^2^ and 0.90 and 1.10 as minimum and maximum efficiency, respectively.

### 2.4. Repeatability, Reproducibility, and Robustness

Assays were carried out with samples of seven animals. The RNA from the blood samples was extracted as previously described and subjected to the RT-qPCR. Repeatability was accessed by testing samples in triplicates and in different days as suggested previously [20]. Tests of reproducibility were performed as described to the repeatability, but by another technician. Robustness was evaluated using another thermocycler, the Rotorgene 3000 (Qiagen, Germany).

### 2.5. Uncertainty of Measurement

Calculation of uncertainty was carried out using the Cts obtained from repeatability and reproducibility assays. Uncertainty value was calculated from the maximum standard deviation values found for each sample in repeatability and reproducibility assays. The uncertainty is stated as the standard uncertainty of measurement multiplied by the coverage factor *k* = 2, which for a normal distribution corresponds to a coverage probability of about 95.45%.

## 3. Results and Discussion

### 3.1. RT-qPCR Amplification and Specificity

The extracted RNAs showed good quality, given that all tested samples were positive on RT-qPCR for beta-actin. Different from that observed by Deng et al. [21], it was not necessary to dilute the RNA from blood samples to obtain positive results on RT-qPCR. Values of threshold cycle (Ct) of the negative controls were not determined and the positive controls had Ct value lower than 35.

Analytical specificity was confirmed through sequencing, where it was observed that the sequence of the amplicons obtained showed high similarity with the CSF virus reference genome available in GenBank (accession number NC 002657.1, *E* value 2*e* − 23 when the sequence was submitted to Blast). Moreover, no cross-reactivity of the CSFV RT-qPCR with the BVDV I and II RNA, ASFV or pseudorabies virus DNA on RT-qPCR was observed.

Hence, the PCR assay was efficient in specifically detecting RNA from CSF virus in all materials from different tissues of infected animals. Furthermore, RNA from CSF virus was not detected in samples of swine originated from the Brazilian CSF-free zone.

### 3.2. Limit of Detection

A blood sample originated from one CSF-infected animal was serially diluted and the diluted samples were tested on RT-qPCR for LD determination. It was possible to detect the viral RNA until the dilution corresponds to 10^−0.8^ TCID_50_/mL, as shown in Table 1. The LD of this RT-qPCR was lower than that described by Agüero et al. [11], who detected on RT-qPCR for* 5*′*NTR* gene the RNA in 0.32 TCID_50_. Hoffmann et al. [12] found different values of LD depending on the viral strain, ranging from 10^−0.75^ TCID_50_/0.1 mL to the Kozlov strain and up to 10^2.5^ TCID_50_/0.1 mL for the Alfort strain.

### 3.3. Repeatability, Reproducibility, Robustness, and Measurement Uncertainty

Results were similarly found for all the three replicates of the dilution and also in the replicates evaluated in three different days. The repeatability, reproducibility, and robustness tests yielded the following values of expanded uncertainty (*U*): *U* = 3.12 when comparing the results of reproducibility and *U* = 1.37 when comparing the results of robustness. Thus it was found that variance is larger when comparing results between technicians than when comparing results obtained from the same equipment and with analyst variation.

Given the results, the following criteria were established in order to validate the analysis: samples were considered positive for CSF when their amplification curves were similar to the positive control curve and exceeded the threshold with Ct values lower that 41, considering the measurement uncertainty *U* = 3.12.

The quality of a laboratorial result is linked to the use of procedures such as validated methods, quality internal controls, participation in interlaboratorial comparison programs, the proper use of certified reference materials, and the compliance with requirements of standards. The understanding of the variables involved is extremely important to produce reliable results. Validation is the confirmation by examination and supply of objective evidence that the particular requirements for a specific use are met. Some of the parameters used in the validation are the uncertainty of the results, the detection limit, and specificity of the method, linearity, repeatability, reproducibility, and robustness [14]. Furthermore, different laboratories or PCR may produce different results. For instance, a ring trial conducted in the framework of the European network of excellence for epizootic disease diagnosis and control demonstrated that some in-house systems had unspecific reactions or suboptimal sensitivity with only a single CSFV genotype [22]. Therefore, it is important to validate and test each PCR in different laboratories and genotypes. In this current study, we were able to reach those standards and specifically confirm the presence of the CSF agent in samples determined as positive for the disease.

## 4. Conclusion

The validation of RT-qPCR performed in this study followed the parameters described in the Manual of Diagnostic Tests and Vaccines for Terrestrial Animals of the OIE [16] and the ISO/IEC 17025:2005 [14]. Since the methods used were standardized, the validation of RT-qPCR shown in this current work meets the standard ISO/IEC 17025:2005 [14]. According to this later standard, when placing a scan of a standardized method and further producing satisfactory results, it can be confirmed that the laboratory is competent for such testing. Furthermore, the RT-qPCR has been demonstrated to be an effective tool for diagnosis of CSF in clinical samples, once it is a fast, sensitive, and specific technique.

## Figures and Tables

**Table 1:** Mean values of Ct and standard deviations of the RT-qPCR for the CSFV in LD determination assays.

TCID_50_/mL	Average of Ct	Deviation
10^4.8^	28.63	0.04
10^3.8^	30.3	0.05
10^2.8^	32.66	0.15
10^1.8^	35.76	0.21
10^0.8^	40.20	3.04
10^−0.2^	Not detected	
